# Occurrence of *bla*_NDM-1_-Positive *Providencia* spp. in a Pig Farm of China

**DOI:** 10.3390/antibiotics11060713

**Published:** 2022-05-25

**Authors:** Wenxin Chen, Zhihong Liu, Hongguang Lin, Jie Yang, Ting Liu, Jiaomei Zheng, Xueming Long, Zhiliang Sun, Jiyun Li, Xiaojun Chen

**Affiliations:** 1 College of Veterinary Medicine, Hunan Agricultural University, Changsha 410128, China; chenwenxin2022@foxmail.com (W.C.); liuzhihongsltg@163.com (Z.L.); linhongguang@stu.hunau.edu.cn (H.L.); yangjie@stu.hunau.edu.cn (J.Y.); liuting1999310@163.com (T.L.); sunzhiliang1965@aliyun.com (Z.S.); 2 Hunan Engineering Technology Research Center of Veterinary Drugs, Hunan Agricultural University, Changsha 410128, China; 3 Changsha Animal and Plant Disease Control Center, Changsha 410003, China; zhengjiaomei3772@163.com; 4 Hunan Provincial Institution of Veterinary Drug and Feed Control, Changsha 410006, China; lxm1250@126.com

**Keywords:** antimicrobial resistance, *bla*_NDM-1_, carbapenem-resistant Enterobacteriaceae, *Providencia*, pig farm

## Abstract

Antibiotics have been extensively used to ensure the productivity of animals on intensive livestock farms. Accordingly, antimicrobial-resistant organisms, which can be transmitted to humans via the food chain, pose a threat to public health. The Enterobacterium antimicrobial resistance gene, *bla*_NDM-1_, is a transmissible gene that has attracted widespread attention. Here, we aimed to investigate the prevalence of Enterobacteriaceae carrying *bla*_NDM-1_ on an intensive pig farm. A total of 190 samples were collected from a pig farm in Hunan Province, China. Resistant isolates were selected using MacConkey agar with meropenem and PCR to screen for *bla*_NDM-1_-positive isolates. Positive strains were tested for conjugation, antimicrobial susceptibility, and whole-genome sequencing. Four *bla*_NDM-1_-positive *Providencia* strains were obtained, and multidrug resistance was observed in these strains. The structure carrying *bla*_NDM-1_ did not conjugate to *E. coli* J53 after three repeated conjugation assays. This suggests that, in intensive farming, attention should be focused on animal health and welfare to reduce the frequency of antibiotic usage. Carbapenem-resistant Enterobacteriaceae in the breeding industry should be included in systematic monitoring programs, including animal, human, and environmental monitoring programs.

## 1. Introduction

Antimicrobial resistance (AMR) is recognized as one of the most serious threats to human and animal health [1,2,3]. These concerns are amplified by the rapid increase in carbapenem-resistant Enterobacteriaceae (CRE), which can carry and spread resistance genes [4]. Infections by CRE are associated with significant morbidity and mortality [5], as well as substantial economic loss.

The mechanisms underlying carbapenem resistance in CRE are complex and are known to involve the production of the enzyme New Delhi metallo-beta-lactamase (NDM-1), a carbapenemase encoded by the *bla*_NDM-1_ gene [6]. Since the first report of a strain of *bla*_NDM-1_-positive *Klebsiella pneumoniae* from India in 2008 [7], many Enterobacteriaceae containing *bla*_NDM-1_ have been reported worldwide [8,9,10]. Several *bla*_NDM-1_-positive isolates belong to the Enterobacteriaceae genus *Providencia*, which consists of several gram-negative opportunistic pathogenic strains [11]. *Providencia* spp. have been reported to cause diarrhea in humans, especially in developing countries, and are a common cause of travel diarrhea [12]. Moreover, *Providencia* spp. have been found in a variety of organisms, including birds, fish, and fruit flies, and can be isolated directly from water [13,14,15,16,17]. The discovery of *bla*_NDM-1_ in *Providencia* is concerning because these bacteria are naturally resistant to tetracycline and colistin, making the clinical treatment of these infections extremely difficult [18]. In the past several years, *bla*_NDM-1_-positive *Providencia* spp. have been found in patients from Peru [19], China [20], and Australia [21], most of whom were hospitalized. These strains are usually isolated from samples of human urine, blood, and sputum [22].

Furthermore, expression of *bla*_NDM-1_ and the presence of its carrier bacteria have been detected in non-clinical samples, such as chickens, pigs, cattle, dogs, cats, swallows, and flies, and transmission of Enterobacteriaceae carrying *bla*_NDM-1_ along the food chain has been reported [4,23]. Thus, there is a precedent for monitoring *bla*_NDM-1-_carrying bacteria in livestock and poultry farms. While investigations on the presence of *bla*_NDM-1_-positive *Providencia* in pigs are scarce [24], the monitoring of zoonotic indicators on pig farms has shown that AMR is common [25]. Therefore, we collected 190 fecal and environmental samples from a large-scale pig farm in Hunan Province, China, to investigate the prevalence of *bla*_NDM-1_-positive bacterial strains. Here, we report the identification of four *bla*_NDM-1_-positive *Providencia* isolates in pigs.

## 2. Results

### 2.1. Identification of bla_NDM-1_-Positive Isolates

Ten strains were screened using meropenem (0.5 µg/mL): three strains were obtained from environmental samples from three different rooms (breeding rooms, pregnancy rooms, and delivery rooms), and six strains were obtained from fecal samples (three from the piglet rooms and three from fattening rooms). PCR analysis of these 10 samples revealed four *bla*_NDM-1_-positive bacterial strains. Three of the four *bla*_NDM-1_-positive strains (20Q122mw, 20Q124mw, and 20Q126mw) were obtained from pig fecal samples collected from the piglet room. The other *bla*_NDM-1_-positive strain (20Q171mw) was obtained from a pig fecal sample collected from a fattening room. Bacterial 16S rRNA sequencing identified strains 20Q122mw and 20Q124mw as *Providencia rettgeri* and strains 20Q126mw and 20Q171mw as *P. stuartii*.

### 2.2. Antimicrobial Susceptibility Profiles and conjugation experiments

The multidrug resistance of the four *bla*_NDM-1_-positive *Providencia strains* was assessed by performing drug sensitivity tests (Table 1). Multidrug-resistant bacteria were defined as those resistant to three or more classes of antibiotics [26]. The minimum inhibitory concentrations (MICs) of florfenicol and colistin against the *bla*_NDM-1_-positive strains exceeded 128 µg/mL, which indicated high resistance. Resistance to florfenicol can be attributed to the presence of the amphenicol resistance gene, floR, in both *P. rettgeri* and *P. stuartii* (Table 2). Meanwhile, resistance to colistin likely corresponds to the natural antimicrobial resistance of *Providencia* since no resistance genes against colistin have been previously found [27]. Although all four *bla*_NDM-1_-positive strains exhibited multidrug resistance, some differences in the resistance profiles between the *P. rettgeri* and *P. stuartii* strains were observed. In particular, a difference was observed in the susceptibility to gentamicin, wherein the *P. stuartii* strains were resistant to gentamicin up to a concentration of 16 µg/mL, whereas the *P. stuartii* strains were sensitive to 0.25 µg/mL gentamicin.

No strains with successful conjugation were obtained after three repeat tests.

### 2.3. WGS Analysis

**Figure 1:**
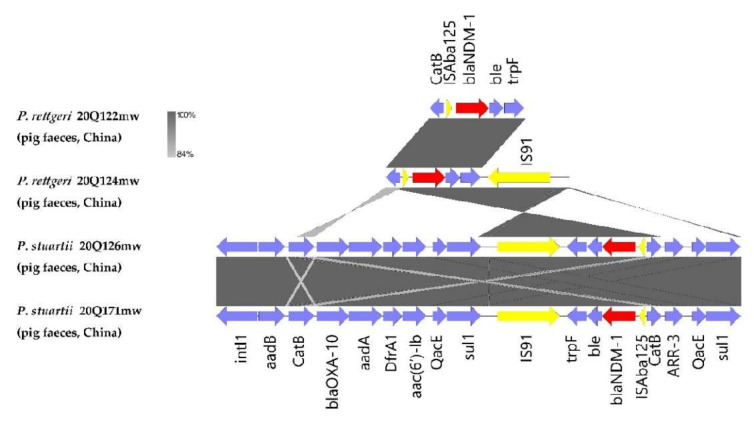
Genomic environment of *bla_NDM-1_* in *P. rettgeri* and *P. stuartii* strains. Genes are represented as arrows, which indicate their transcription orientations and relative lengths.

**Figure 2:**
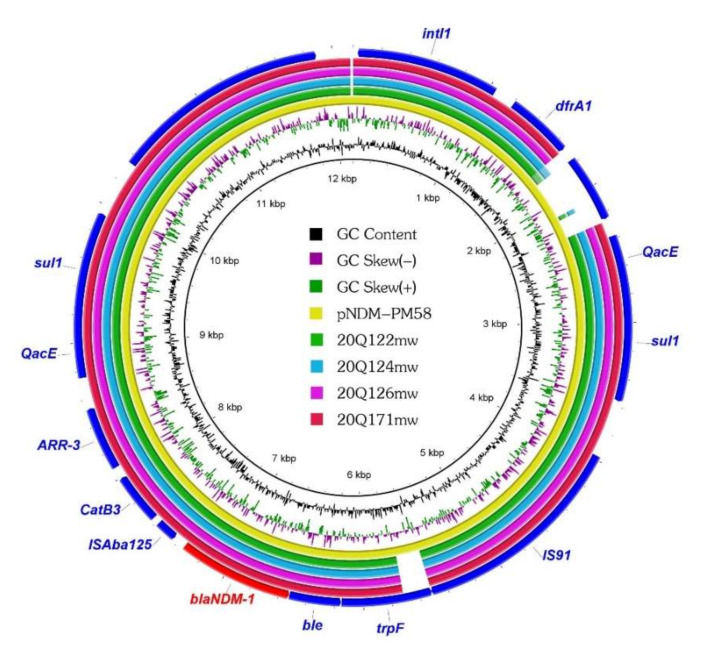
Comparison of the whole-genome sequences of *Providencia* spp. strains with the sequence of plasmid pNDM-PM58 (GenBank accession number KP662515.1).

The distribution of drug resistance genes in the four *Providencia* strains was analyzed by using whole genome sequencing. In addition to the beta-lactam resistance genes *bla*_NDM__-1_, *bla*_OXA-10_, and *bla*_TEM-116_, the four *bla*_NDM-1_-positive strains carried several common resistance genes (Table 2). In the two *P. rettgeri* strains, resistance genes against aminoglycosides (*aph(3’)-Ia, aadA1, aac(6’)-Ib-cr*, and *aadA2*), macrolides (*msr(E)* and *mph(E)*), trimethoprims (*dfrA1* and *dfrA12*), and sulfonamides (*sul1*) were identified. Interestingly, we detected more resistance genes in the *P. stuartii* strains than in the *P. rettgeri* strains; these included resistance genes against aminoglycosides (*aph(6)-Id, aph(3’)-Ia, aadA1, aac(6’)-Ib3, aph(3‘)-Ib, aac(6’)-Ib-cr*, and *ant(2’’)-Ia*), macrolides (*msr(E)* and *mph(E)*), trimethoprims (*dfrA1*), and sulfonamides (*sul1* and *sul2*). Some of these resistance genes were distributed around *bla*_NDM-1_. *Bla*_NDM__-1_ in all four strains were found on the same fragment containing the *QacE-sul1-IS91-trpF-ble-bla_NDM-1_-ISAba125-CatB-ARR-3-QacE-sul1* cassette (Figure 1 and Figure 2), suggesting that the transmission of this fragment occurred in the pig farm. 

In order to facilitate the comparison between human and pig isolates, a *Proteus mirabilis* plasmid isolated from the urine of a patient at the Zhengzhou University Hospital in China was analyzed (Figure 2) [28]. The *Proteus mirabilis’* plasmid, named *pNDM-PM58* (GenBank accession number KP662515.1), was 12,146 bp in size. We found that the *QacE-sul1-IS91-trpF-ble-bla_NDM-1_-ISAba125-CatB-ARR-3-QacE-sul1* cassette array of *Providencia* was the same as that of *pNDM*-*PM58*, except for the *trpF* gene, wherein the *pNDM-PM58* had a complete *trpF* gene than *Providencia*. Moreover, similar to the *bla*_NDM-1_-positive *Providencia strains*, *P. mirabilis* exhibited resistance to meropenem and some cephalosporins. However, *pNDM-PM58* obtained from humans exhibited significant resistance to amikacin (at >64 µg/mL), whereas the four *bla*_NDM-1_-positive *Providencia* strains were sensitive to amikacin (at 4 µg/mL).

## 3. Discussion

To the best of our knowledge, this study is the first to identify *bla*_NDM-1_ in *Providencia* spp. isolated from pigs. These strains were obtained from pig fecal samples from a pig farm in China, specifically from piglet and fattening rooms. As members of the Enterobacteriaceae family, the discovery of *bla*_NDM-1_-positive *Providencia* in food-producing animals is of concern. Enterobacteriaceae harboring *bla*_NDM-1_ are resistant to many antibacterial agents and cause problems in the treatment of many bacterial infections in animals and humans. Genetic segments containing *bla*_NDM-1_ can conjugate with different bacteria via mobile genetic elements, leading to the spread of multidrug resistance [29,30,31]. Although the four *bla*_NDM-1_-positive *Providencia* strains identified in this study had inserted sequences around *bla*_NDM-1_, they did not conjugate with *E. coli* J53 after three repeated conjugation assays. This might be due to preferential conjugation effects between bacteria; for example, the insert sequences of the IS30 family were observed to preferentially insert into the inverted repeat sequence of similar regions than with themselves [32]. It was possible that the conditions of our conjugation tests did not meet the conditions for transfer or that transfer to *E. coli* J53 was not compatible with the *bla*_NDM-1_-positive *Providencia* strains. 

Among the various bacterial genera present in the samples collected in this study, *bla*_NDM-1_ was only detected in *Providencia* strains. Therefore, *Providencia* strains were probably the dominant population for *bla*_NDM-1_ transmission in this pig farm. These isolates were collected from different rooms (piglet and fattening rooms) and identified as different species (*P. rettgeri* and *P. stuartii*); however, all isolates harbored the same *bla*_NDM-1_ segment. This suggests that horizontal gene transfer is a probable route of transmission in this farm. Furthermore, whole genome sequencing analyses identified consistent clusters of highly genetically-related isolates (20Q122mw and 20Q124mw; 20Q126mw and 20Q171mw), suggesting the possibility of *bla*_NDM-1_ vertical transmission.

On intensive pig farms, personnel disinfection is usually strictly enforced to ensure the health of animals, and meropenem use is not allowed. However, *bla*_NDM-1_-positive *Providencia* strains were detected. Thus, it is possible that the presence of *bla*_NDM-1_ on this pig farm might have originated from *Providencia* harboring *bla*_NDM-1_ in wild hosts, such as wild birds, flies, and mice. Preventing exposure to wild host species may help prevent the transfer of *bla*_NDM-1_-carrying strains to pigs.

In addition, we noted some interesting observations while analyzing the genetic environment near *bla*_NDM-1_. Compared to the pNDM-PM58 plasmid of *P. mirabilis* isolated from a human sample, a similar segment (*QacE-sul1-IS91-trpF-ble-bla*_NDM-1_*-ISAba125*) was found in the *bla*_NDM-1_-positive *Providencia* strains. While a similar segment shared by different bacteria is not necessarily proof of horizontal gene transfer as they might originate from different sources [33], this segment that contains *bla*_NDM-1_ has the potential to spread between humans and animals.

## 4. Materials and Methods

In December 2020, 190 fecal (*n* = 137) or environmental (*n* = 53) samples were collected from a pig farm in Hunan Province, China. Pig fecal samples were collected from the following areas: breeding rooms (*n* = 28), pregnancy rooms (*n* = 27), delivery rooms (*n* = 44), piglet rooms (*n* = 26), and fattening rooms (*n* = 12). Environmental samples, including from pig drinking water, sewage, and dirt, were collected from the following areas: breeding rooms (*n* = 9), pregnancy rooms (*n* = 11), delivery rooms (*n* = 18), piglet rooms (*n* = 9), and fattening rooms (*n* = 6). All samples were collected using sterile swabs and were then suspended in 1 mL phosphate-buffered saline. These samples were stored in iceboxes and transported to the laboratory at the end of sampling. MacConkey agar (Landbridge, Beijing, China) supplemented with 0.5 µg/mL meropenem (Meilun Biotechnology Co. Ltd., Dalian, China) and vancomycin (30 µg/mL, Meilun Biotechnology Co. Ltd., Dalian, China) was used to screen for antibiotic-resistant bacterial strains in the samples. PCR and electrophoresis assays were performed to verify whether the filtered isolates contained *bla*_NDM-1_ [34]. The 16S rRNA sequencing was performed to confirm the presence of the bacterial species by Tsingke Biotechnology Company (Changsha, China).

The antimicrobial sensitivity of *bla*_NDM-1_-positive isolates for 11 antibiotics was assessed. The agar dilution method was used for meropenem, amikacin, florfenicol, tigecycline, gentamicin, ciprofloxacin, cefotaxime, ceftiofur, tetracycline, and piperacillin-tazobactam (Meilun Biotechnology Co. Ltd., Dalian, China), whereas the broth microdilution method was used for colistin (Meilun Biotechnology Co. Ltd., Dalian, China), according to the guidelines of the Clinical and Laboratory Standards Institute (CLSI) (https://clsi.org, 1 October 2021). *Escherichia coli* ATCC 25922 was used as the control strain.

Subsequently, *bla*_NDM-1_-positive strains were used as donor bacteria and sodium azide-tolerant *E. coli* J53 as receptor bacteria. The donor and receptor bacteria were mixed at a ratio of 1:3 in LB broth (Landbridge, Beijing, China), and the mixture was spread onto microporous membranes affixed to Mueller Hinton agar (Landbridge, Beijing, China) at 37 °C for 18 h. The colonies on the membranes were then diluted in LB broth and evenly coated onto MacConkey agar plates containing 1 µg/mL meropenem and 400 µg/mL sodium azide. The cultures were incubated at 37 °C for 18 h. Then, the strains were filtered using these cultures. Conjugation assays were performed in triplicate.

DNA from all *bla*_NDM-1_-positive isolates was extracted using the TIANamp Bacteria DNA Kit (Tiangen Biotech Co., China), according to the manufacturer’s instructions. WGS of 20Q122mw, 20Q124mw, and 20Q171mw was performed using the Illumina HiSeq sequencing platform (Illumina, San Diego, CA, USA) by Annoroad Gene Technology (Beijing, China). Meanwhile, genomic DNA sequencing of strain 20Q126mw was performed using the Nanopore MinION (100-fold average read depth). Raw sequences were assembled using SPAdes 3.11 (Bankevich et al., 2012) and annotated using RAST (http://rast.nmpdr.org/, accessed on 8 January 2022). AMR genes were searched in the Center for Genomic Epidemiology database (www.genomicepidemiology.org, accessed on 8 January 2022). The Basic Local Alignment Search Tool (BLAST) of the National Center for Biotechnology Information (NCBI) (https://blast.ncbi.nlm.nih.gov/Blast.cgi, accessed on 16 January 2022) was used to analyze the alignments of similar sequences. The genetic environment of *bla*_NDM-1_ was investigated using Easyfig 2.2.5 [35]. The BLAST Ring Image Generator (BRIG 0.9516) was used to generate a comparative genomic circle map [36]. 

## 5. Conclusions

In this study, four strains of *bla*_NDM-1_-positive *Providencia* spp. were found in the feces of swine. These four strains carried the same segments of *bla*_NDM-1_ and were isolated from different farming rooms. Furthermore, the segments were similar to that of *Proteus mirabilis*, which was isolated from a patient in Zhengzhou, China, thereby indicating that these segments of *bla*_NDM-1_ can be transmitted between humans and animals. In order to reduce the risk of AMR transmission, the conditions in pig farms should be further optimized to minimize the use of antibiotics. AMR monitoring of intensive farms should be maintained to keep humans and animals safe.

## Figures and Tables

**Table 1:** Susceptibility of *bla*_NDM-1_-positive *Providencia* strains to antimicrobial agents.

Strains	Sites	Species	Source	MIC (µg/mL)										
				MEM	AMK	FFC	CL	TGC	GEN	CIP	CTX	CEF	TCY	PTZ
20Q122mw	Piglet room	*P. rettgeri*	Pig feces	4	4	>128	>128	1	0.25	4	16	16	2	32
20Q124mw	Piglet room	*P. rettgeri*	Pig feces	4	4	>128	>128	1	0.25	4	16	16	2	32
20Q126mw	Piglet room	*P. stuartii*	Pig feces	4	4	>128	>128	2	16	4	16	32	4	32
20Q171mw	Fattening room	*P. stuartii*	Pig feces	4	4	>128	>128	2	16	4	16	32	4	32

MIC, minimum inhibitory concentration; MEM, meropenem; AMK, amikacin; FFC, florfenicol; CL, colistin; TGC, tigecycline; GEN, gentamicin; CIP, ciprofloxacin; CTX, cefotaxime; CEF, ceftiofur; TCY, ceftiofur; PTZ, piperacillin-tazobactam.

**Table 2:** Genetic characterization of carbapenem-resistant *Providencia* strains.

Strains	Source	Species	Sequencing Platforms	Size	GC Content	Antibiotic Resistance Genes
20Q122mw	Pig feces	*P. rettgeri*	Illumina Hiseq	4,612,974 bp	40.3%	*bla*NDM-1, *bla*TEM-116, *msr(E)*, *aph(3*′*)-Ia*, *aadA1*, *aac(6*′*)-Ib-cr*, *aadA2*, *dfrA1*, *dfrA12*, *sul1*, *tet(D)*, *mph(E)*, *qnrA1*, *ARR-3*, *qacE*, *floR*, *catB3*
20Q124mw	Pig feces	*P. rettgeri*	Illumina Hiseq	4,607,625 bp	40.3%	*bla*NDM-1, *msr(E)*, *aac(6*′*)-Ib-cr*, *aph(3*′*)-Ia*, *aadA2*, *aadA1*, *tet(D)*, *qnrA1*, *sul1*, *dfrA12*, *dfrA1*, *ARR-3*, *mph(E)*, *qacE*, *catB3*, *floR*
20Q126mw	Pig feces	*P. stuartii*	Nanopore minION	4,712,152 bp	40.7%	*bla*NDM-1, *bla*OXA-10, *dfrA1*, *sul1*, *sul2*, *aph(6)-Id*, *aac(6*′*)-Ib3*, *aac(6*′*)-Ib-cr*, *aadA1*, *ant(2″)-Ia*, *aph(3*′*)-Ia*, *aph(3″)-Ib*, *ARR-3, mph(E)*, *msr(E)*, *qnrD1*, *tet(B)*, *qacE*, *catB8, floR*, *catB3*, *lnu(G)*
20Q171mw	Pig feces	*P. stuartii*	Illumina Hiseq	4,665,324 bp	40.6%	*bla*NDM-1, *bla*OXA-10, *bla*TEM-116, *ant(2″)-Ia*, *aadA1*, *aph(3*′*)-Ia*, *aac(6*′*)-Ib-cr*, *aph(3″)-Ib*, *aph(6)-Id*, *aac(6’)-Ib3, dfrA1*, *sul1*, *sul2*, *ARR-3*, *mph(E)*, *msr(E)*, *qnrD1*, *tet(B)*, *qacE*, *catB8*, *catB3*, *floR*, *lnu(G)*

## Data Availability

All genome sequences have been deposited in the GenBank database under the BioProject accession number SAMN26356030, SAMN26356031, SAMN26356032 and SAMN26356033.

## References

[1] Brinkac L., Voorhies A., Gomez A., Nelson K.E. (2017). The Threat of Antimicrobial Resistance on the Human Microbiome. Microb. Ecol..

[2] Sartelli M., Weber D.G., Ruppé E., Bassetti M., Wright B.J., Ansaloni L., Catena F., Coccolini F., Abu-Zidan F.M., Coimbra R. (2016). Antimicrobials: A global alliance for optimizing their rational use in intra-abdominal infections (AGORA). World J. Emerg. Surg..

[3] Sharma C., Rokana N., Chandra M., Singh B.P., Gulhane R.D., Gill J.P.S., Ray P., Puniya A.K., Panwar H. (2018). Antimicrobial Resistance: Its Surveillance, Impact, and Alternative Management Strategies in Dairy Animals. Front. Vet. Sci..

[4] Wang Y., Zhang R., Li J., Wu Z., Yin W., Schwarz S., Tyrrell J.M., Zheng Y., Wang S., Shen Z. (2017). Comprehensive resistome analysis reveals the prevalence of NDM and MCR-1 in Chinese poultry production. Nat. Microbiol..

[5] Logan L.K., Weinstein R.A. (2017). The Epidemiology of Carbapenem-Resistant Enterobacteriaceae: The Impact and Evolution of a Global Menace. J. Infect. Dis..

[6] Wu W.F.Y., Tang G., Qiao F., McNally A., Zong Z. (2019). Ndm metallo-β-lactamases and their bacterial producers in health care settings. Clin. Microbiol. Rev..

[7] Yong D., Toleman M.A., Giske C.G., Cho H.S., Sundman K., Lee K., Walsh T.R. (2009). Characterization of a new metallo-beta-lactamase gene, bla(ndm-1), and a novel erythromycin esterase gene carried on a unique genetic structure in klebsiella pneumoniae sequence type 14 from india. Antimicrob. Agents Chemother..

[8] Li J., Lan R., Xiong Y., Ye C., Yuan M., Liu X., Chen X., Yu D., Liu B., Lin W. (2014). Sequential Isolation in a Patient of Raoultella planticola and Escherichia coli Bearing a Novel ISCR1 Element Carrying blaNDM-1. PLoS ONE.

[9] Dortet L., Poirel L., Nordmann P. (2014). Worldwide Dissemination of the NDM-Type Carbapenemases in Gram-Negative Bacteria. BioMed Res. Int..

[10] Phan H.T.T., Stoesser N., Maciuca I., Toma F., Szekely E., Flonta M., Hubbard A.T.M., Pankhurst L., Do T., Peto T. (2017). Illumina short-read and MinION long-read WGS to characterize the molecular epidemiology of an NDM-1 Serratia marcescens outbreak in Romania. J. Antimicrob. Chemother..

[11] Oliveira H., Pinto G., Hendrix H., Noben J.P., Gawor J., Kropinski A.M., Łobocka M., Lavigne R., Azeredo J. (2017). A lytic providencia rettgeri virus of potential ther-apeutic value is a deep-branching member of the t5virus genus. Appl. Environ. Microbiol..

[12] Di H., Liang S., Li Q., Shi L., Shima A., Meng H., Yan H., Yamasaki S. (2018). Providencia in retail meats from Guangzhou, China and Osaka, Japan: Prevalence, antimicrobial resistance and characterization of classes 1, 2 and 3 integrons. J. Vet. Med. Sci..

[13] Andolfo G., Schuster C., Gharsa H.B., Ruocco M., Leclerque A. (2021). Genomic analysis of the nomenclatural type strain of the nema-tode-associated entomopathogenic bacterium providencia vermicola. BMC Genomics..

[14] Galac M.R., Lazzaro B.P. (2012). Comparative genomics of bacteria in the genus providencia isolated from wild drosophila melanogaster. BMC Genom..

[15] Interaminense J.A., Nascimento D.C.O., Ventura R.F., Batista J.E.C., Souza M.M.C., Hazin F.H.V., Pontes-Filho N.T., Lima-Filho J. (2010). Recovery and screening for antibiotic susceptibility of potential bacterial pathogens from the oral cavity of shark species involved in attacks on humans in Recife, Brazil. J. Med. Microbiol..

[16] Mnif B., Ktari S., Chaari A., Medhioub F., Rhimi F., Bouaziz M., Hammami A. (2013). Nosocomial dissemination of providencia stuartii isolates carrying bla oxa-48, bla per-1, bla cmy-4 and qnra6 in a tunisian hospital. J. Antimicrob. Chemother..

[17] Yuan C., Wei Y., Zhang S., Cheng J., Cheng X., Qian C., Wang Y., Zhang Y., Yin Z., Chen H. (2020). Comparative Genomic Analysis Reveals Genetic Mechanisms of the Variety of Pathogenicity, Antibiotic Resistance, and Environmental Adaptation of Providencia Genus. Front. Microbiol..

[18] O’Hara CM B.F., Miller J.M. (2000). Classification, identification, and clinical significance of proteus, providencia, and morganella. Clin. Microbiol. Rev..

[19] Lezameta L., Cuicapuza D., Dávila-Barclay A., Torres S., Salvatierra G., Tsukayama P., Tamariz J. (2020). Draft genome sequence of a new delhi metallo-β-lactamase (ndm-1)-producing providencia stuartii strain isolated in Lima, Peru. Microbiol. Resour. Announc..

[20] Shen S., Huang X., Shi Q., Guo Y., Yang Y., Yin D., Zhou X., Ding L., Han R., Yu H. (2022). Occurrence of NDM-1, VIM-1, and OXA-10 Co-Producing Providencia rettgeri Clinical Isolate in China. Front. Cell. Infect. Microbiol..

[21] Wailan A.M., Paterson D.L., Kennedy K., Ingram P.R., Bursle E., Sidjabat H.E. (2016). Genomic characteristics of ndm-producing entero-bacteriaceae isolates in australia and their blandm genetic contexts. Antimicrob Agents Chemother..

[22] Xia S., Xiao S., Zhang Q., Zhao Q., Lin B., Huang Z., Zhuo C., Chen R. (2013). Emergence of carbopenem resistant providencia rettgeri and its resistance mechanisms. J. Third Mil. Med. Univ..

[23] Köck R., Daniels-Haardt I., Becker K., Mellmann A., Friedrich A.W., Mevius D., Schwarz S., Jurke A. (2018). Carbapenem-resistant Enterobacteriaceae in wildlife, food-producing, and companion animals: A systematic review. Clin. Microbiol. Infect..

[24] Rui P., Tian T., Liu X., Zhang D., Yang C., Liu X., Song T., Meng Y., Ma Z. (2017). Isolation and identification of providencia rettgeri which caused diarrhea in piglets. Chin. J. Vet. Sci..

[25] European Food Safety A., European Centre for Disease Control (2019). The european union summary report on antimicrobial re-sistance in zoonotic and indicator bacteria from humans, animals and food in 2017. EFSA J..

[26] Zhao Q., Berglund B., Zou H., Zhou Z., Xia H., Zhao L., Nilsson L.E., Li X. (2021). Dissemination of blandm-5 via incx3 plasmids in car-bapenem-resistant enterobacteriaceae among humans and in the environment in an intensive vegetable cultivation area in eastern china. Environ. Pollut..

[27] Hayakawa K., Marchaim D., Divine G.W., Pogue J.M., Kumar S., Lephart P., Risko K., Sobel J.D., Kaye K.S. (2012). Growing prevalence of Providencia stuartii associated with the increased usage of colistin at a tertiary health care center. Int. J. Infect. Dis..

[28] Qin S., Qi H., Zhang Q., Zhao D., Liu Z.-Z., Tian H., Xu L., Xu H., Zhou M., Feng X. (2015). Emergence of Extensively Drug-Resistant Proteus mirabilis Harboring a Conjugative NDM-1 Plasmid and a Novel Salmonella Genomic Island 1 Variant, SGI1-Z. Antimicrob. Agents Chemother..

[29] Zhu X., Zhang Y., Shen Z., Xia L., Wang J., Zhao L., Wang K., Wang W., Hao Z., Liu Z. (2021). Characterization of NDM-1-Producing Carbapenemase in *Proteus mirabilis* among Broilers in China. Microorganisms.

[30] Xiang T., Chen C., Wen J., Liu Y., Zhang Q., Cheng N., Wu X., Zhang W. (2020). Resistance of Klebsiella pneumoniae Strains Carrying blaNDM–1 Gene and the Genetic Environment of blaNDM–1. Front. Microbiol..

[31] Tang B., Chang J., Cao L., Luo Q., Xu H., Lyu W., Qian M., Ji X., Zhang Q., Xia X. (2019). Characterization of an NDM-5 carbapenemase-producing Escherichia coli ST156 isolate from a poultry farm in Zhejiang, China. BMC Microbiol..

[32] Olasz F., Kiss J., Arini A., Arber W. (1997). Terminal inverted repeats of insertion sequence is30 serve as targets for transposition. J. Bacteriol.

[33] Weber R.E., Pietsch M., Fruhauf A., Pfeifer Y., Martin M., Luft D., Gatermann S., Pfennigwerth N., Kaase M., Werner G. (2019). Is26-mediated transfer of bla ndm-1 as the main route of resistance transmission during a polyclonal, multispecies outbreak in a german hospital. Front. Microbiol..

[34] Poirel L., Walsh T.R., Cuvillier V., Nordmann P. (2011). Multiplex PCR for detection of acquired carbapenemase genes. Diagn. Microbiol. Infect. Dis..

[35] Sullivan M.J., Petty N.K., Beatson S.A. (2011). Easyfig: A genome comparison visualizer. Bioinformatics.

[36] Alikhan N.-F., Petty N.K., Ben Zakour N.L., Beatson S.A. (2011). BLAST Ring Image Generator (BRIG): Simple prokaryote genome comparisons. BMC Genom..

